# Interhospital Transfer Patients With Necrotizing Soft Tissue Infections: An Evaluation of the Role for Pretransport Operation

**DOI:** 10.1002/wjs.70546

**Published:** 2026-09-02

**Authors:** Hans V. Strobl, Diane N. Haddad, Renee Bicaba, Renee I. Tristano, Phillip M. Dowzicky, Abid D. Khan, Timothy P. Plackett, Justin S. Hatchimonji

**Affiliations:** ^1^ Section of Trauma and Acute Care Surgery University of Chicago Medicine Chicago Illinois USA; ^2^ Department of Surgery Loyola University Medical Center Maywood Illinois USA

**Keywords:** acute care surgery, necrotizing soft tissue infection, transfer

## Abstract

**Background:**

Early debridement is the cornerstone of treatment for necrotizing soft tissue infections (NSTI), but patients are frequently transferred for management. We described practice patterns, hypothesizing that pre‐transfer NSTI debridement is associated with improved outcomes.

**Methods:**

We analyzed data from the Healthcare Cost and Utilization Project (HCUP) State Inpatient Databases (SID) and State Emergency Department Databases (SEDD) for Massachusetts, Wisconsin, and Colorado in 2022. NSTI patients and the subset undergoing operation before transfer (PRE) were identified. The primary outcome was mortality. We performed multivariable logistic regression adjusting for demographics, weekend transfer, severe sepsis or septic shock, and Charlson comorbidity index (CCI). Secondary outcomes were length of stay (LOS) and hospital charges.

**Results:**

We identified 243 transferred NSTI patients. Mean age (SD) was 56.7 (14.5) years, and 74 (30.5%) were female. There were 127 referring and 52 receiving centers, with a wide range of case volume and PRE rates. Seventy‐seven (31.7%) patients were PRE; there was no difference in rates of sepsis between these patients and the remainder (41.6% vs. 37.4%, *p* = 0.531). Mortality with and without pre‐transfer operation was 7/77 (9.1%) versus 10/166 (6.0%), with aOR = 1.62 for PRE, (*p* = 0.38). Pre‐ and post‐transfer median LOS were longer for PRE (4 vs. 0 days at the referring hospital, *p* < 0.001; 15 vs. 10 days at the receiving hospital, *p* = 0.01), and PRE had higher median charges before ($66,214.50 vs. $11,227.50, *p* < 0.001) and after transfer ($161,615 vs. $89,728, *p* = 0.012).

**Conclusion:**

Among transferred NSTI patients, mortality did not differ between PRE and the remainder of the cohort. PRE patients had longer hospital stays and higher healthcare charges, possibly due to worse physiology or more complex wounds.

## Graphical abstract

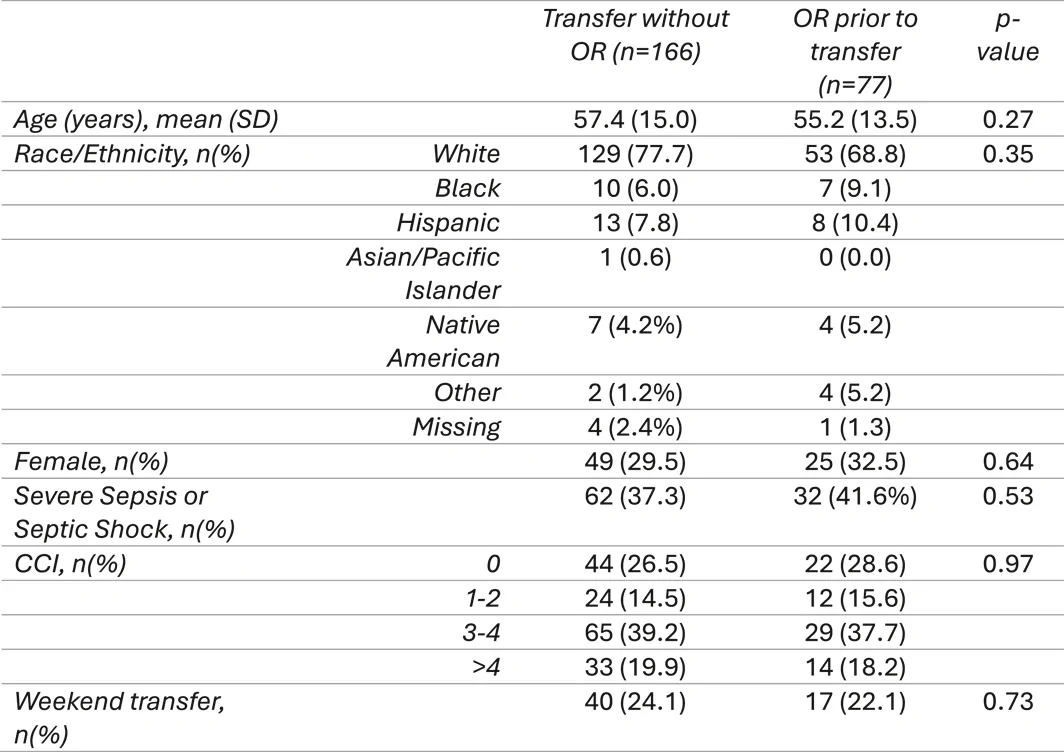

## 1 Background

Necrotizing soft tissue infections (NSTI) are rapidly progressive infections that traverse the epidermis, dermis, and subcutaneous tissue, with extension along fascial planes and frequent involvement of underlying muscle. These infections often lead to sepsis and are highly lethal without prompt intervention. Early surgical debridement remains the cornerstone of treatment—although guidelines exist that separate septic (1–2 hours) and nonseptic (6 hours) patients, it is generally accepted to perform the debridement as soon as possible [1, 2]. Despite substantial advances in critical care and surgical management, NSTIs continue to carry a pooled mortality in the US of approximately 13% in the modern era [3, 4].

Given the high mortality and the need for rapid intervention, timely and accurate diagnosis is essential. However, NSTIs are rare, limiting the firsthand experience of most providers. Incidence estimates are widely variable, but are likely fewer than 30,000 per year in the United States [5, 6]. Diagnosis is further complicated by the heterogeneity in clinical presentation. Although physical exam findings such as crepitus or necrosis and imaging evidence of subcutaneous gas are strongly indicative of NSTI, these hallmark features are absent in many patients, limiting their diagnostic utility. Laboratory abnormalities including hyponatremia, leukocytosis, and elevated C‐reactive protein have been incorporated into the Laboratory Risk Indicator for Necrotizing Fasciitis (LRINEC) score; however, this tool has not been validated in large prospective studies [7]. Consequently, operative exploration remains the gold standard for definitive diagnosis.

NSTI management is complex due to its high morbidity and mortality, the frequent need for repeated debridement, intensive critical care requirements, and the involvement of multiple subspecialties and rehabilitation services. This leads many patients to be transferred to tertiary and quaternary centers for comprehensive care. Although well intentioned and often necessary, interfacility transfer can introduce delays in initiating the early surgical debridement these patients require.

The existing literature comparing outcomes in transferred patients with those who undergo immediate debridement is mixed [8, 9, 10, 11, 12, 13]. Among patients transferred with a diagnosis of NSTI, we sought to determine whether there was a mortality difference between those who underwent operative debridement before versus after transfer. We hypothesized that patients who underwent surgery prior to transfer would have a lower mortality rate.

## 2 Methods

We obtained administrative claims data from the Healthcare Cost and Utilization project (HCUP) State Inpatient Databases (SID) and State Emergency Department Databases (SEDD). The SEDD encompasses all visits to a hospital‐owned emergency department (ED) that do not result in hospitalization [14]. The SID encompasses > 95% of discharges from all inpatient stays (including those admitted via an ED) [15]. The data for this project were from Massachusetts, Wisconsin, and Colorado, for the year 2022, using both the SID and SEDD. These states were felt to represent a spectrum of geography and offer necessary data fields for this research question. 2022 was the most recent year available at the time of analysis.

First, transfer patients were identified. For each of the three states, we screened data for inpatient‐inpatient transfers (SID to SID) and ED‐inpatient transfers (SEDD to SID). Patients transferred ED to ED and subsequently admitted appear in the SID. HCUP documentation recommends visits be linked using a unique anonymized identifier (VisitLink) combined with an assigned variable denoting anonymized time points. This variable, DaysToEvent, begins with a random number from which all events (admissions) for the year are measured. Thus, a given patient's transfer date can be defined as the date of discharge from the index hospital = DaysToEvent+(length of stay), as well as the date of admission to the receiving hospital (DaysToEvent for that stay). Patients for whom these numbers were equal, for whom the index discharge disposition was transfer to another acute care hospital, and for whom the admission source for the second stay was another acute hospital were defined as transfer patients. Of note, Massachusetts employs different VisitLinks for the SEDD and SID, meaning we could not track ED‐inpatient transfers in this state; only inpatient–inpatient.

Next, from the pool of transfer patients, we identified those with a diagnosis of NSTI. To do this, we used International Classification of Diseases (ICD‐10) codes A48.0 (“gas gangrene”), M72.6 (“necrotizing fasciitis”), and N49.3 (“Fournier's gangrene”), in concordance with prior literature [6, 16]. Prior work has also included the code I96, which we chose to exclude because it indicates “gangrene not otherwise specified”, which might include lower extremity dry gangrene, approached with a different level of urgency. The diagnosis codes both prior to and following transfer were screened. Those with an NSTI diagnosis made up the cohort for this study.

The primary exposure was operation prior to transfer. This was defined by HCUP flag for a major operating room procedure during the index hospitalization. The primary outcome of interest was mortality. We performed unadjusted comparisons using chi‐squared tests and logistic regression adjusting for covariates. Covariates included age, weekend transfer, sex, sepsis or septic shock as defined by ICD‐10 codes R65.20 and R65.21, and Charlson Comorbidity Index, which we calculated from diagnoses designated as present on admission [17]. These covariates were chosen based on clinical suspicion and known risk factors; however, out of concern for possible model overspecification we repeated the analysis using a reduced number of covariates. We additionally described length of stay (LOS) pre‐ and post‐transfer, as well as total charges. Comparisons of length of stay and charges were made using Wilcoxon rank‐sum tests. Analyses were conducted using Stata 19.5 (College Station, TX). This study was deemed exempt by the Institutional Review Board at our institution.

## 3 Results

We identified 243 patients that were transferred between hospitals with a diagnosis of NSTI. Mean age (SD) was 56.7 (14.5), and 74 (30.5%) were female. There were 127 referring centers and 52 receiving centers in the cohort. Volume of transferred NSTI patients varied between 1 and 25 among the receiving hospitals. Among receiving hospitals with more than 10 transferred patients for the year, rates of pretransfer operation ranged from 0% to 69.6%. Volume of transferred NSTI patients varied between 1 and 8 among transferring hospitals, with widely variable practices on pretransfer operation. Of the 243 total patients, 77 of them (31.7%) underwent operation prior to transfer. Patients undergoing operation prior to transfer did not differ significantly from those being transferred without operation, in age, race, sex, sepsis, CCI, or weekend transfer (Table 1).

**TABLE 1:** Baseline characteristics of patients undergoing operation prior to transfer versus not.

		Transfer without OR (*n* = 166)	OR prior to transfer (*n* = 77)	*p*‐value
Age (years), mean (SD)		57.4 (15.0)	55.2 (13.5)	0.27
Race/ethnicity, *n* (%)	White	129 (77.7)	53 (68.8)	0.35
	Black	10 (6.0)	7 (9.1)	
	Hispanic	13 (7.8)	8 (10.4)	
	Asian/pacific islander	1 (0.6)	0 (0.0)	
	Native american	7 (4.2%)	4 (5.2)	
	Other	2 (1.2%)	4 (5.2)	
	Missing	4 (2.4%)	1 (1.3)	
Female, *n* (%)		49 (29.5)	25 (32.5)	0.64
Severe sepsis or septic shock, *n* (%)		62 (37.3)	32 (41.6%)	0.53
CCI, *n* (%)	0	44 (26.5)	22 (28.6)	0.97
	1–2	24 (14.5)	12 (15.6)	
	3–4	65 (39.2)	29 (37.7)	
	> 4	33 (19.9)	14 (18.2)	
Weekend transfer, *n* (%)		40 (24.1)	17 (22.1)	0.73

Abbreviations: CCI, Charlson comorbidity index; OR, operating room; SD, standard deviation.

On unadjusted analysis, there was no statistically significant difference in mortality based on whether patients underwent operation prior to transfer or not. Mortality was 10/166 (6.0%) in those without pretransfer operation and 7/77 (9.1%) in those with one (*p* = 0.38). We also did not detect a statistically significant difference in mortality on logistic regression analysis adjusted for age, weekend transfer, female sex, sepsis, and CCI (OR 1.6 with pre‐transfer operation, *p* = 0.375) (Table 2). Race was not included in this analysis because a small proportion of patients were missing race data entirely (*n* = 5, 2.1%), and some smaller categories (Asian/Pacific Islander and “Other”) predicted survival perfectly and thus resulted in observations being removed from the cohort (*n* = 7, 2.9%). We did not feel that the benefit of including this variable warranted losing 5% of the cohort. To create our simplified model, we tested the above covariates independently with an a priori plan to include those with *p* < 0.2. Based on this, age (OR 1.03, *p* = 0.139) and sepsis (OR 4.21, *p* = 0.009) were included. The simplified model yielded results similar to the expanded model, with OR for pretransfer operation 1.7 (*p* = 0.330).

**TABLE 2:** Multivariable regression analysis, mortality.

		OR	95% CI	*p*‐value
Pre‐transfer operation		1.62	0.56–4.67	0.375
Age		1.03	0.99–1.07	0.183
Female		2.10	0.72–6.08	0.172
CCI	0	(Ref)		
	1–2	2.21	0.43–11.37	0.342
	3–4	1.11	0.25–5.07	0.889
	> 4	2.42	0.50–11.77	0.275
Severe sepsis/Septic shock		4.82	1.59–14.64	0.006
Weekend transfer		1.16	0.37–3.67	0.804

Abbreviations: CCI, Charlson comorbidity index; CI, confidence interval; OR, odds ratio.

Median [interquartile range (IQR)] pre‐ and post‐transfer length of stay are shown for each group in Table 3. Both pre‐ and post‐transfer lengths of stay were longer in the group undergoing operation prior to transfer; pre‐transfer operation patients stayed a median of 4 days at the index hospital and 15 days at the receiving hospital. Accordingly, median charges, also shown in Table 3, were higher both pre‐ and post‐transfer in the group who underwent pretransfer operation ($66,214.50 and $161,615, respectively).

**TABLE 3:** Length of stay and total charges pre‐ and post‐transfer for patients with and without pre‐transfer operation. Total charges provided in US dollars.

	Pre‐transfer operation	No pre‐transfer operation	*p*‐value
Pre‐transfer LOS, median [IQR]	4 [0, 9]	0 [0, 1]	< 0.001
Post‐transfer LOS, median [IQR]	15 [8, 28]	10 [5, 20]	0.011
Pre‐transfer charges, median [IQR]	66,214.50 [44,537.50, 128,456]	11,227.50 [8,002, 22,099]	< 0.001
Post‐transfer charges, median [IQR]	161,615 [65,733, 297,742]	89,728 [48,410, 203,891]	0.012

Abbreviations: IQR, interquartile range; LOS, length of stay.

## 4 Discussion

Among patients transferred with a diagnosis of NSTI, we examined whether mortality differed between those who underwent initial operative debridement prior to transfer versus not. We found no statistically significant difference in mortality between these groups. Additionally, pre‐ and post‐transfer lengths of stay were both longer among patients who underwent operation prior to transfer, and median charges were correspondingly higher in this group.

Prior literature primarily compares outcomes between patients directly admitted to tertiary centers (i.e., from the emergency department) versus those who are transferred. These prior studies have reported mixed results. Specifically, Holena et al. and Khoury et al. have compared transfer patients to nontransfer patients, demonstrating lower mortality among nontransfer patients [8, 9]. However, another study found no significant difference in mortality between these groups [10]. Furthermore, one study identified a higher mortality rate among direct admissions, although this finding was not statistically significant. In that study, transferred patients were also significantly more likely to proceed to the operating room within 6 hours of arrival than those who were directly admitted, suggesting differences in early operative management [11]. An analysis of NSQIP data demonstrated reduced mortality among direct admissions [12]. However, institutions participating in NSQIP are typically tertiary care centers [18], which may lead to better outcomes due to the availability of resources and surgical personnel. Finally, a study of NSTI patients treated at burn centers reported higher mortality among transferred patients, although it was unclear whether transferred patients displayed more deranged physiology to start [13]. Although valuable, these findings address a fundamentally different question than the one being asked here.

To our knowledge, no prior studies have focused exclusively on transferred patients, making our approach unique. The current study restricts the cohort to transferred patients and examines outcomes based on whether the patient underwent a debridement prior to transfer. Importantly, our analysis includes patients treated at both tertiary referral centers and referring community hospitals, seeking to shed light on the issue of pre‐transfer debridement at less‐resourced, non‐tertiary centers. In addition to finding no statistically significant difference in mortality, we found longer pre‐ and post‐transfer lengths of stay, as well as higher charges, in patients who underwent operation prior to transfer.

The reasons for the above findings—longer length of stay and costs—is not entirely clear, but there are two primary reasonable hypotheses. First, it is possible that the patients who underwent operation prior to transfer were more physiologically deranged, hence the longer length of stay despite no statistically significant difference in mortality. If there were a benefit to pretransfer debridement, this would bias our results towards the null. We have attempted to account for sepsis in our model, but the lack of physiologic data precludes detailed assessment of sepsis severity at presentation. The State Inpatient Databases (SID) and State Emergency Department Databases (SEDD) are administrative datasets and do not include granular clinical variables such as vital signs or laboratory values; consequently, the extent of end‐organ dysfunction could not be determined. A second potential explanation for the length of stay and cost findings is that some proportion of patients undergoing operation prior to transfer require transfer not because of their critical care needs, but for more advanced reconstruction. The administrative nature of these data sources poses the same problem here; although it would be helpful to know who was seen by reconstructive surgeons and/or the number and extent of reconstructive operations, we lack this data.

There are additional limitations to discuss here. Because the data field used here is a binary flag for whether the patient got a “major OR procedure,” we lack information on the number of operations undertaken prior to transfer, or their timing. Furthermore, it is theoretically possible that some NSTI patients underwent a major OR procedure for some other reason, leading to a misclassification bias; however, it seems hard to imagine a clinical scenario in which a patient is diagnosed with an NSTI and undergoes some other operation without undergoing a debridement. An additional, informative outcome to measure would have been time to operation in these patients; unfortunately, we lack that data. Similarly, adjustment for center‐ and surgeon‐level volume, experience, and training was not possible. Finally, the relatively small sample size results in limited statistical power, increasing the risk of a type II error and reducing our ability to detect a true difference in outcomes between groups.

Notwithstanding these limitations, this study provides some evidence relevant to a situation that is frequently encountered among patients with an infrequent problem. Although we did not demonstrate an increase in mortality without a pre‐transfer operation, there was also no statistically significant increase in mortality with one, despite the aforementioned suggestion that these patients were likely in worse condition preoperatively. It is unlikely to be harmful to the patient for a general surgeon to perform an expeditious basic debridement prior to transfer; this would be our recommendation. However, it is clear that further evidence is needed on this topic, in the form of a study with more detailed physiologic information to address confounding and more detailed timing data to determine whether waiting for transfer actually prolongs time to the OR, and whether this has an ultimate impact on mortality.

## 5 Conclusion

Our study demonstrated no statistically significant difference in mortality between NSTI transfer patients undergoing pretransfer operation versus not. Patients who underwent operative intervention prior to transfer experienced longer pre‐ and post‐transfer lengths of stay, and correspondingly higher median charges.

## Author Contributions

**Hans V. Strobl:** conceptualization, writing – original draft. **Diane N. Haddad:** writing – original draft. **Renee Bicaba:** writing – review and editing. **Renee I. Tristano:** writing – original draft. **Phillip M. Dowzicky:** writing – review and editing. **Abid D. Khan:** writing – review and editing. **Timothy P. Placket:** writing – review and editing. **Justin S. Hatchimonji:** conceptualization, data curation, formal analysis, methodology, resources, supervision, writing – original draft.

## Funding

The authors have nothing to report.

## Conflicts of Interest

The authors declare no conflicts of interest.

## Data Availability

The data that support the findings of this study are available from the Healthcare Cost and Utilization Project (HCUP). Restrictions apply to the availability of these data, which were used under license for this study. Data are available from the author(s) with the permission of the Healthcare Cost and Utilization Project (HCUP).
